# Synthesis of Exosome-Based Fluorescent Gold Nanoclusters for Cellular Imaging Applications

**DOI:** 10.3390/ijms22094433

**Published:** 2021-04-23

**Authors:** Eun Sung Lee, Byung Seok Cha, Seokjoon Kim, Ki Soo Park

**Affiliations:** Department of Biological Engineering, College of Engineering, Konkuk University, 120 Neungdong-ro, Gwangjin-gu, Seoul 05029, Korea; afish94@konkuk.ac.kr (E.S.L.); cbs934@konkuk.ac.kr (B.S.C.); ghjghy@konkuk.ac.kr (S.K.)

**Keywords:** exosome, gold nanocluster, cellular imaging, fluorescence imaging

## Abstract

In recent years, fluorescent metal nanoclusters have been used to develop bioimaging and sensing technology. Notably, protein-templated fluorescent gold nanoclusters (AuNCs) are attracting interest due to their excellent fluorescence properties and biocompatibility. Herein, we used an exosome template to synthesize AuNCs in an eco-friendly manner that required neither harsh conditions nor toxic chemicals. Specifically, we used a neutral (pH 7) and alkaline (pH 11.5) pH to synthesize two different exosome-based AuNCs (exo-AuNCs) with independent blue and red emission. Using field-emission scanning electron microscopy, energy dispersive X-ray microanalysis, nanoparticle tracking analysis, and X-ray photoelectron spectroscopy, we demonstrated that AuNCs were successfully formed in the exosomes. Red-emitting exo-AuNCs were found to have a larger Stokes shift and a stronger fluorescence intensity than the blue-emitting exo-AuNCs. Both exo-AuNCs were compatible with MCF-7 (human breast cancer), HeLa (human cervical cancer), and HT29 (human colon cancer) cells, although blue-emitting exo-AuNCs were cytotoxic at high concentrations (≥5 mg/mL). Red-emitting exo-AuNCs successfully stained the nucleus and were compatible with membrane-staining dyes. This is the first study to use exosomes to synthesize fluorescent nanomaterials for cellular imaging applications. As exosomes are naturally produced via secretion from almost all types of cell, the proposed method could serve as a strategy for low-cost production of versatile nanomaterials.

## 1. Introduction

Fluorescent metal nanoclusters (NCs), composed of several to hundreds of atoms with a sub-nanometer size, are formed by using biomolecules as a template [1]. Because biomolecule-templated metal NCs exhibit excellent fluorescence properties and require less-toxic conditions for their synthesis than organic fluorophores, they have been widely used for various purposes, such as bioimaging, sensing, and drug delivery [2,3]. Representative examples of these types of nanomaterial are gold, silver, copper, and platinum NCs that are synthesized via their interactions with proteins or nucleic acids [4]. AuNCs are generally formed on a protein template, while AgNCs and CuNCs are formed on a nucleic acid template [5,6,7]. Bovine serum albumin (BSA) was the first protein used to produce AuNCs, which have been widely employed for cellular imaging and biomolecular sensing [8,9]. Furthermore, other protein templates such as insulin, lysozyme, and transferrin have been utilized for the synthesis of fluorescent AuNCs [10,11,12]. Many researchers have continued to investigate new biomolecular templates for the synthesis of fluorescent metal NCs [13].

Exosomes are small vesicles (50‒150 nm in size) that are secreted by almost cells through the endosomal pathway [14]. As exosomes encapsulate biomolecules, such as proteins, nucleic acids, and lipids, that originate from parental cells, they play an important role in intra/inter-cellular communication and contribute to cancer metastasis [15,16,17,18]. In recent years, many research efforts have been focused on deciphering the information provided by exosomes so as to enable non-invasive cancer diagnoses [19,20]. It has been reported that exosomes contain various proteins, including origin-specific markers (EpCAM and MUC-1) and general markers (HSP 70, TSG101, CD63, and CD81) [21,22,23]. In addition, exosomes with stable phospholipid bilayer structures have been used as drug delivery vehicles [24,25]. Based on their homing properties, exosomes can even target specific cells without the need for the presence of targeting ligands [26].

In this study, we used exosomes to develop a new strategy for the synthesis of fluorescent NCs. As a proof of concept, we synthesized AuNCs—which are known to be the least toxic and most chemically stable among the various fluorescent NCs [27,28]—using proteins containing amino acids such as tyrosine and cysteine, which reduce Au(III) to Au(I) and form Au-S bonds to stabilize AuNCs, respectively [29,30,31]. We expected AuNCs to be effectively formed using exosomes containing various proteins. Using the proposed strategy, we prepared two different exosome-based AuNCs (exo-AuNCs), one with a blue emission and the other with a red emission, by simply adjusting the pH. After characterizing the prepared exo-AuNCs with different analytical tools, we analyzed their toxicity using different cell lines (MCF-7, HeLa, and HT29). Finally, we used cellular imaging to analyze the different cell lines and assess the compatibility of the exo-AuNCs with other membrane-staining dyes. This study on the use of exosomes as fluorescence imaging probes may broaden the potential utility of exosome-based NCs as tools for applications in diagnosis, therapy, and drug delivery.

## 2. Results and Discussion

### 2.1. Synthesis of Exo-Gold Nanoclusters (AuNCs)

**Figure 1:**
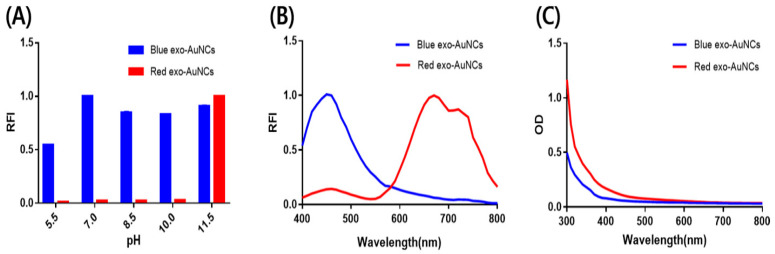
Synthesis of exo-gold nanoclusters (AuNCs)**.** (**A**) The effect of pH on the formation of blue- and red-emitting exo-AuNCs. The relative fluorescence intensity (RFI) was calculated by dividing the fluorescence intensity at different pH values (5.5, 7.0, 8.5, 10.0, and 11.5) by the maximum fluorescence intensity at emission wavelengths of 460 nm (blue) and 670 nm (red); both blue- and red-emitting exo-AuNCs were excited at 360 nm. (**B**) Relative fluorescence emission spectra of blue- and red-emitting exo-AuNCs at an excitation wavelength of 360 nm. The RFI was calculated by dividing the fluorescence intensity at different wavelengths by the maximum fluorescence intensity. (**C**) Ultraviolet–visible (UV-Vis) absorbance spectra of exo-AuNCs. The OD in Y-axis indicates the optical density. All experiments were performed in triplicate, and the data are displayed as the mean ± SD.

As the tyrosine residue exhibits a strong reducing ability at pH values higher than its pKa (*ca.* 10) and as the thiol group of the cysteine residue binds tightly to the Au ions [29,30,31], we expected that exosomes containing various proteins with tyrosine and cysteine residues would mediate the formation of fluorescent AuNCs. First, we investigated the effect of pH on the formation of exo-AuNCs. As shown in Figure 1A,B, blue-emitting exo-AuNCs with a maximum emission intensity at 460 nm were formed at various pH values, while red-emitting exo-AuNCs with a maximum emission intensity at 670 nm were only formed at pH 11.5. Importantly, blue- and red-emitting exo-AuNCs with high fluorescence signals were generated only when both exosomes and Au ions were present (Figure S1), and their fluorescence emission spectra matched well with the one of BSA-AuNCs (Figure S2), indicating that the protein in exosome is the major component for the synthesis of exo-AuNCs. The fluorescence intensity of the red-emitting exo-AuNCs was much higher (*ca.* 10×) than that of the blue-emitting exo-AuNCs, in accordance with previous reports [32]. In addition, using an absorbance analysis, we confirmed that Au nanoparticles larger than the AuNCs were not formed, as indicated by the lack of absorbance peaks around 500 nm (Figure 1C) [33]. Overall, we succeeded in using exosome templates for the synthesis of exo-AuNCs that emitted fluorescence of different wavelengths.

### 2.2. Optimization of Conditions for Exo-AuNC Synthesis

**Figure 2:**
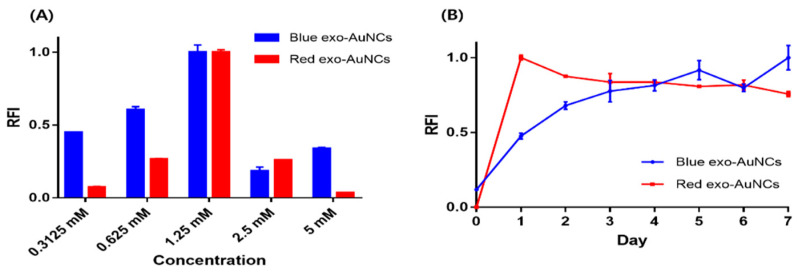
Optimization of conditions for synthesis of exo-AuNCs. (**A**) The effect of Au ion concentration on the formation of blue- and red-emitting exo-AuNCs. The exosome concentration was fixed at 6.25 mg/mL. The relative fluorescence intensity (RFI) was calculated by dividing the fluorescence intensity at different Au ion concentrations (0.3125, 0.625, 1.25, 2.5, and 5 mM) by the maximum fluorescence intensity at emission wavelengths of 460 nm (blue) and 670 nm (red); both blue- and red-emitting exo-AuNCs were excited at 360 nm. (**B**) The effect of reaction time on the formation of blue- and red-emitting exo-AuNCs. The RFI was calculated by dividing the fluorescence intensity at different reaction times (0 to 7 days) by the maximum fluorescence intensity at emission wavelengths of 460 nm (blue) and 670 nm (red); the red dot represents the RFI at 670 nm; both blue- and red-emitting exo-AuNCs were excited at 360 nm.

We assumed that the concentration of Au ions and the reaction time, in addition to the pH value (Figure 1A), would be critical for the formation of exo-AuNCs. As shown in Figure 2A, both blue- and red-emitting exo-AuNCs exhibited their highest fluorescence intensities at an Au ion concentration of 1.25 mM. Furthermore, the results in Figure 2B show that the fluorescence of the blue-emitting exo-AuNCs gradually increased up to the third day, after which the fluorescence became saturated, while the red-emitting exo-AuNCs reached their highest fluorescence intensities after 1 day, beyond which the fluorescence intensities decreased. The reduction in fluorescence after 1 day was attributed to the high pH used to facilitate the formation of red-emitting exo-AuNCs, which can damage exosomes. Based on these optimization experiments, 1.25 mM Au ions and 3 and 1 days for blue- and red-emitting exo-AuNCs, respectively, were chosen as the conditions for further experiments.

### 2.3. Characterization of Exo-AuNCs

**Figure 3:**
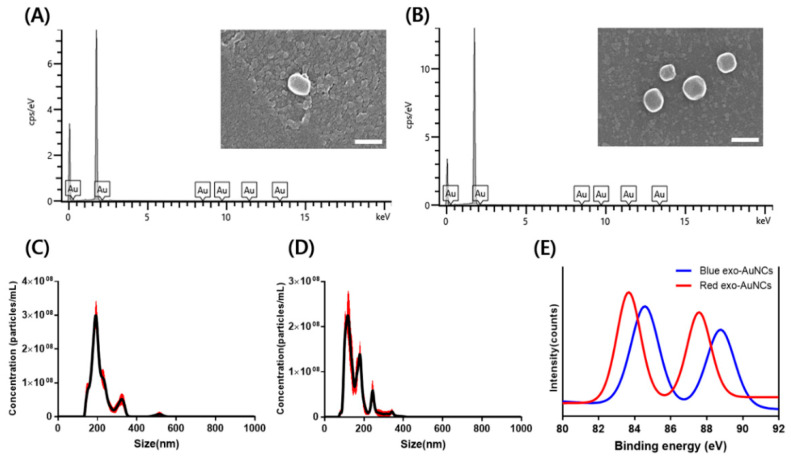
Characterization of exo-AuNCs. (**A**,**B**) Field-emission scanning electron microscopy (FE-SEM) images and energy dispersive X-ray microanalysis results of blue- (left; **A**) and red-emitting (right; B) exo-AuNCs. The scale bar in the FE-SEM images is 200 nm. (**C**,**D**) Nanoparticle tracking analysis of blue- (left; **C**), and red-emitting (right; **D**) exo-AuNCs. The red shadow indicates the standard deviation of the mean. (**E**) X-ray photoelectron spectroscopy spectra of blue- and red-emitting exo-AuNCs.

Under the optimal conditions (blue: 6.25 mg/mL exosomes, 1.25 mM Au ions, 37 °C for 3 days at pH 7; red: 6.25 mg/mL exosomes, 1.25 mM Au ions, 37 °C for 1 day at pH 11.5), blue- and red-emitting exo-AuNCs were synthesized and then characterized using different analytical tools. First, the exo-AuNCs were imaged using FE-SEM to confirm that the unique shape of the exosomes was maintained. As shown in Figure 3A,B, the exosomes maintained their vesicle shape, with a size of less than 200 nm in both blue- and red-emitting exo-AuNCs. These results confirmed that the synthesis conditions for exo-AuNCs did not alter the physical properties of the exosome, enabling the maintenance of the unique shape of the exosomes. Next, through EDX analysis, it was confirmed that Au ions were present in both the blue- and red-emitting exo-AuNCs (Figure 3A,B). In addition, NTA was used to assess the size distribution and concentration of exosomes. The results shown in Figure 3C,D indicate that the size and number of exosomes were 136.2 ± 3.1 nm and 2.01 × 10^10^ particles/mL, respectively, in the blue-emitting exo-AuNCs, and 116 ± 5.9 nm and 1.63 × 10^10^ particles/mL, respectively, in the red-emitting exo-AuNCs. These findings were in accordance with previously reported results [33,34]. Furthermore, we investigated the number of blue- and red-emitting exo-AuNCs using X-ray photoelectron spectroscopy (XPS) [34]. As shown in Figure 3E, both blue- and red-emitting exo-AuNCs had similar Au 4f_5/2_ and 4f_7/2_ binding energy peaks with the protein-templated AuNCs in previous report [35]. Thus, we assumed that blue- and red-emitting exo-AuNCs contain 8 (Au_8_NC) and 25 (Au_25_NC) Au atoms, respectively. Importantly, the 4f_5/2_ and 4f_7/2_ binding energy peaks of blue-emitting AuNCs were 88.48 eV and 84.28 eV, whereas those of red-emitting AuNCs were 87.58 eV and 83.68 eV, respectively, demonstrating that blue-emitting AuNCs was relatively smaller than red-emitting AuNCs. In addition, the binding energy of blue-emitting exo-AuNCs is attributed to Au (0) only, and that of red-emitting exo-AuNCs is attributed to 90% of Au (0) and 10% of Au(I) (Figure S3). These results were consistent with the fact that red-emitting AuNCs was composed of an Au (0) core surrounded by Au (I) and had a smaller binding energy peak than that of blue-emitting AuNCs [32].

### 2.4. Cytotoxicity of Exo-AuNCs

**Figure 4:**
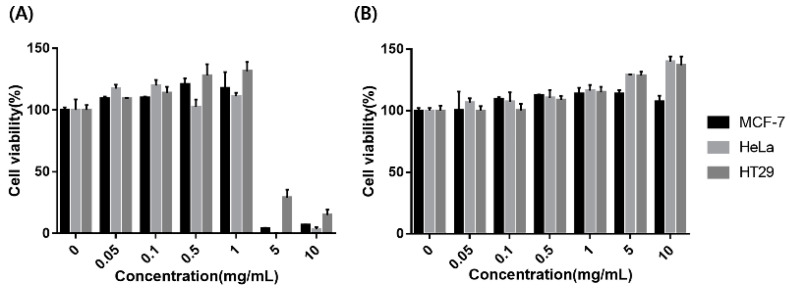
Cytotoxicity of exo-AuNCs. (**A**) Blue-emitting exo-AuNCs. (**B**) Red-emitting exo-AuNCs. MCF-7, HeLa, and HT29 cells were incubated with different concentrations of exo-AuNCs (0.05, 0.1, 0.5, 1, 5, and 10 mg/mL) for 24 h.

We evaluated the cytotoxicity of exo-AuNCs using an MTT (3-(4,5-dimethylthiazol-2-yl)-2,5-diphenyltetrazolium bromide) assay. Blue- and red-emitting exo-AuNCs at different concentrations (0.05, 0.1, 0.5, 1, 5, and 10 mg/mL) were used to treat different cell lines. As shown in Figure 4A, the blue-emitting exo-AuNCs at concentrations up to 1 mg/mL did not exhibit cytotoxicity, but at concentrations of 5 and 10 mg/mL, they were highly toxic to all three cell lines. Conversely, the red-emitting exo-AuNCs at concentrations of up to 10 mg/mL did not exhibit any cytotoxicity. Interestingly, all three cell types grew better in the presence of the red-emitting exo-AuNCs at high concentrations, implying that these compounds had a beneficial effect on cell growth [36]. We assumed that the difference in cytotoxicity between the blue- and red-emitting exo-AuNCs could be attributed to the difference in their sizes (8 versus 25 Au atoms for blue- and red-emitting exo-AuNCs, respectively). Overall, the red-emitting exo-AuNCs were less toxic to cells and exhibited excellent biocompatibility.

### 2.5. Cellular Imaging Using Exo-AuNCs

**Figure 5:**
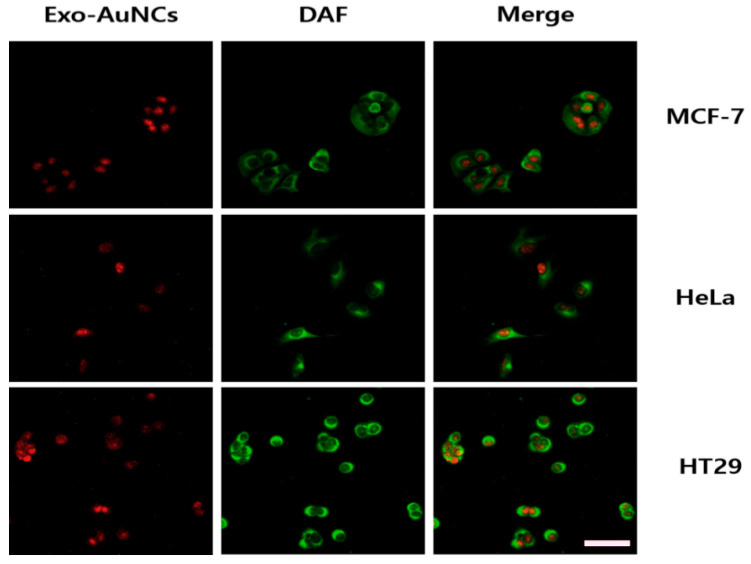
Fluorescence imaging using exo-AuNCs. Scale bar: 50 µm. The cell membrane was counterstained with 5-dodecanoylaminofluorescein (500 µM). The concentration of red-emitting exo-AuNCs was 3.125 mg/mL.

We used the red-emitting exo-AuNCs for cellular imaging because they had better fluorescence properties (a large Stokes shift with excitation and emission wavelengths at 360 and 670 nm, respectively) and biocompatibility than the blue-emitting ones. After the cells were incubated with the red-emitting exo-AuNCs, the cell membranes were counterstained with 5-dodecanoylaminofluorescein (DAF). In all three cell lines, the red-emitting exo-AuNCs effectively stained the nuclei (Figure 5), as indicated by the bright red color, and were compatible with DAF. These results were consistent with previous reports that fluorescence nanomaterials with a positive charge can stain the cell nucleus [37]. Our findings demonstrated that fluorescent exo-AuNCs may be useful as new bio-imaging probes.

## 3. Materials and Methods

### 3.1. Materials

We used the following materials and equipment: fetal bovine serum (FBS; Sigma-Aldrich, St. Louis, MO, USA), ExoQuick-TC reagent (SBI, Los Angeles, CA, USA), phosphate-buffered saline (PBS; Biosesang, Gyeonggi-do, Korea), gold(III) chloride trihydrate (HAuCl_4_; Sigma-Aldrich, St. Louis, MO, USA), deionized sterile water (DW; Bioneer, Daejeon, Korea), Macrosep Advance Centrifugal Device (30 kDa; Pall Corporation, Washington, NY, USA), Micro BCA^TM^ protein assay kit (Thermo Scientific, Waltham, MA, USA), Dulbecco’s modified eagle medium/high glucose (DMEM; HyClone, Logan, UT, USA), Roswell park memorial institute 1640 medium (RPMI; HyClone, Logan, UT, USA), Minimum essential media (MEM; Welgene, Gyeongsangbuk-do, Korea), penicillin-streptomycin (Gibco, Brooklyn, NY, USA), Dulbecco’s phosphate buffered saline(DPBS; Sigma-Aldrich, St. Louis, MO, USA), 3-(4,5-dimethylthiazol-2-yl)-2,5-diphenyltetrazolium bromide (MTT; Invitrogen, Carlsbad, CA, USA), and 5-dodecanoylaminofluorescein (DAF; Invitrogen, Carlsbad, CA, USA).

### 3.2. Isolation of Exosomes

The exosomes were isolated from FBS using the ExoQuick-TC reagent according to the manufacturer’s protocol. In brief, FBS was first centrifuged at 3000× *g* for 15 min on the Macrosep Advance Centrifugal Device. Next, the retentate containing the concentrated exosomes was transferred to a new sterile tube and mixed with the ExoQuick-TC reagent at a 5:1 ratio. After overnight incubation at 4 °C, the mixture was centrifuged at 1500× *g* for 30 min. The supernatant was removed without disturbing the pellet formed at the bottom of the tube. Finally, the pellet containing the exosomes was washed twice with 1× PBS, resuspended in PBS, and then stored at −20 °C until further use.

### 3.3. Synthesis of Exo-AuNCs

First, 1 mL of exosomes (6.25 mg/mL) in 1 × PBS (pH 7.4) and 1 mL of HAuCl_4_ (1.25 mM) in DW were mixed at room temperature (i.e., 25 °C) for 5 min. Exosome concentration was quantified using the Micro BCA^TM^ protein assay kit. Next, the pH of the mixture was adjusted using NaOH, and the mixture was incubated in a shaking incubator for 3 days at 37 °C and 200 rpm.

### 3.4. Characterization of Exo-AuNCs

The Spectramax iD5 Multi-Mode Microplate Reader (Molecular Device, San Jose, CA, USA) was used to measure the absorbance and fluorescence spectra of the prepared exo-AuNCs. After the exo-AuNCs were synthesized, field emission scanning electron microscopy (FE-SEM; SU8010, Hitachi, Tokyo, Japan) was used to examine the shape of the exosomes, and energy dispersive X-ray (EDX) microanalysis was used to confirm the formation of the exo-AuNCs [38]. Nanoparticle tracking analysis (NTA; NS300, Malvern-Panalytical, Malvern, Worcestershire, UK) was used to measure the concentration and size of the exosomes. X-ray photoelectron spectroscopy (XPS; K-Alpha, Thermo Fisher Scientific, Waltham, MA, USA) was used to confirm the number of Au particles in the blue- and red-emitting exo-AuNCs.

### 3.5. Cell Culture

The MCF-7 (human breast cancer), HeLa (human cervical cancer), and HT29 (human colon cancer) cell lines were purchased from Korean Cell Line Bank (KCLB, Seoul, Korea) and cultured in an atmosphere of 5% CO_2_ an incubator at 37 °C. MCF-7 cells were cultured in RPMI 1640 medium supplemented with 10% FBS and 1% penicillin-streptomycin. HeLa and HT29 cells were cultured under the same conditions as the MCF-7 cells, except the RPMI 1640 medium was replaced with MEM and DMEM, respectively.

### 3.6. MTT (3-(4,5-Dimethylthiazol-2-yl)-2,5-diphenyltetrazolium Bromide) Assay

First, the MCF-7, HeLa, and HT29 cells were seeded in RPMI 1640, MEM, and DMEM, respectively, in 96-well culture plates (1 × 10^4^ cells per well). After 24 h of incubation at 37 °C in an atmosphere of 5% CO_2_, the cells were washed twice with DPBS, transferred to fresh media containing different concentrations of exo-AuNCs (0.05, 0.1, 0.5, 1, 5, and 10 mg/mL), and then incubated for 24 h. The concentrations of exo-AuNCs were based on the exosome concentrations quantified using the Micro BCA^TM^ protein assay kit. Next, 20 µL of MTT solution (5 mg/mL) was added, and the cells were incubated for 4 h. Finally, 10% sodium dodecyl sulfate (SDS) was added, and the cells were incubated for another 24 h. The resulting absorbance signal at 595 nm was measured and used to estimate cell viability (%).

### 3.7. Cellular Imaging

First, the MCF-7, HeLa, and HT29 cells were seeded in black 96-well culture plates (1 × 10^4^ cells per well) and incubated for 24 h. After they were washed three times with DPBS and fixed with 4% paraformaldehyde for 15 min, the cells were incubated with exo-AuNCs (3.125 mg/mL) for 6 h. The cells were then washed three times with DW. Next, the cells were counterstained with DAF (500 µM) for 15 min and washed three times with DW. Finally, images were obtained using fluorescence microscopy (KI-2000F; Korea Lab Tech, Gyeonggi-do, Republic of Korea) under the following conditions: filter cube 1 (excitation: 330–385 nm; barrier: 420 nm) was used to image exo-AuNCs with excitation and emission wavelengths of 360 nm and 670 nm, respectively; while filter cube 2 (excitation: 450–480 nm; barrier: 515 nm) was used to image DAF with excitation and emission wavelengths of 460 nm and 520 nm, respectively.

## 4. Conclusions

In summary, we developed a new method for the synthesis of fluorescent AuNCs, using an exosome template. Our method differs from that used in most exosome-related studies, which have focused on biomarker analysis or drug delivery. By optimizing the reaction conditions, we succeeded in preparing blue- and red-emitting exo-AuNCs with emission wavelengths of 460 nm and 670 nm, respectively, which were successfully characterized using various analytical tools. Notably, the fluorescent exo-AuNCs were produced by simply incubating a mixture of exosomes and Au ions at room temperature, without the need for harsh experimental conditions or toxic chemicals. Furthermore, the red-emitting exo-AuNCs had a large Stokes shift, exhibited no cellular toxicity, and were successfully used for nuclear staining in various cell lines. Considering the numerous unique features of exosomes, we believe that exo-AuNCs would represent a versatile platform for bio-imaging. This study may serve as the basis for the synthesis of other nanomaterials.

## Data Availability

The data supporting the results are included in this published article and its Supplementary Information Files.

## Supplementary Materials

The following are available online at https://www.mdpi.com/article/10.3390/ijms22094433/s1, Figure S1: Synthesis of exo-AuNCs, Figure S2: Fluorescence Spectra of BSA-AuNCs and exo-AuNCs, Figure S3: XPS spectra of blue- and red-emitting exo-AuNCs.
