# Supplementary material for: Synthesis of Exosome-Based Fluorescent Gold Nanoclusters for Cellular Imaging Applications
Source: Int J Mol Sci. 2021 Apr 23;22(9):4433. doi: 10.3390/ijms22094433 (PMC8122875; doi:10.3390/ijms22094433)
Supplement: Supplementary file 1 [file ijms-22-04433-s001.zip › ijms-1189879-supplementary.pdf]

Supplementary Material

Synthesis of exosome-based fluorescent gold nanoclusters for cellular imaging applications

Eun Sung Lee, Byung Seok Cha, Seokjoon Kim, and Ki Soo Park

Department of Biological Engineering, College of Engineering, Konkuk University, 120 Neungdong-ro, Gwangjin-gu, Seoul 05029, Korea

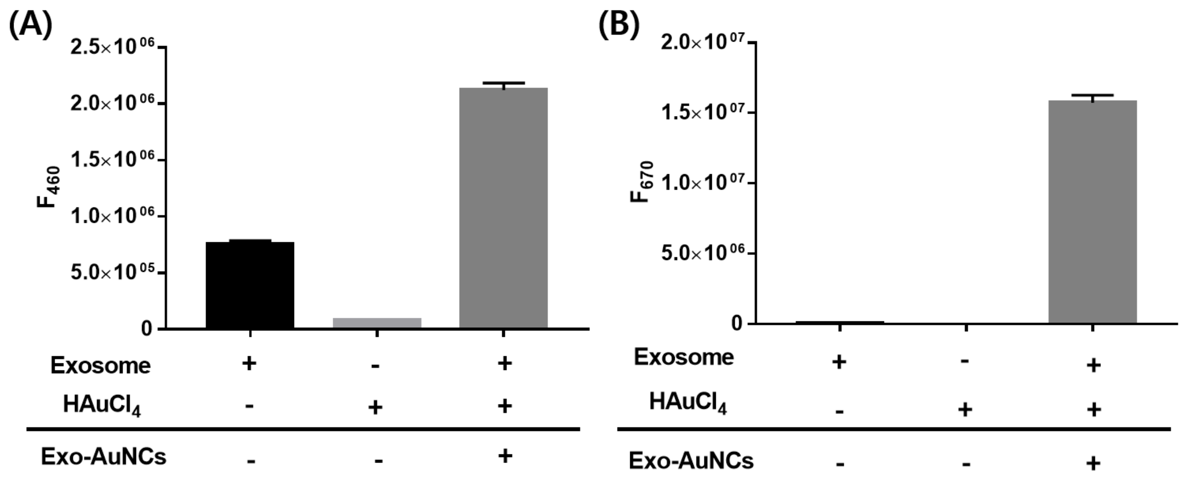

Figure S1. Synthesis of exo-AuNCs. (A) and (B) are the results of blue- and red-emitting exo-AuNCs, respectively. The excitation wavelength for both (A) and (B) was 360 nm. F_{460} and F_{670} indicate the fluorescence emission intensity at 460 nm (A) and 670 nm (B), respectively.

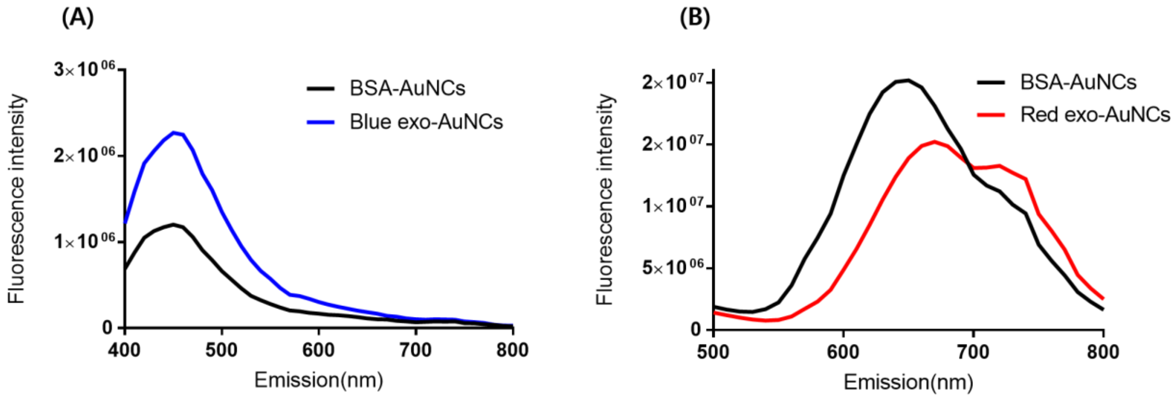

Figure S2. Fluorescence spectra of BSA-AuNCs and exo-AuNCs. (A) Fluorescence spectra of blue-emitting BSA- and exo-AuNCs. (B) Fluorescence spectra of red emitting BSA- and exo-AuNCs. The excitation wavelength for both (A) and (B) was 360 nm. Both BSA and exosome was used at the same protein concentration of 6.25 mg/mL for the synthesis of AuNCs.

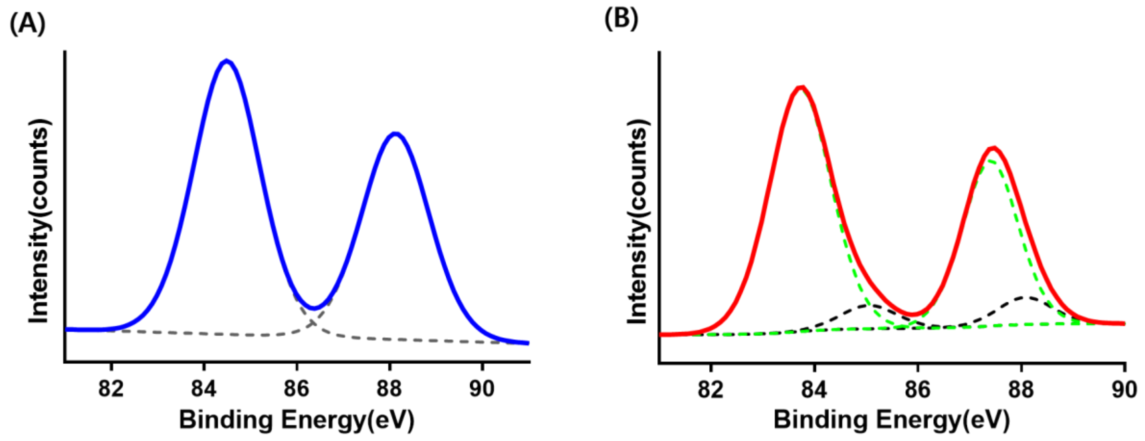

Figure S3. XPS spectra of blue- and red-emitting exo-AuNCs. (A) XPS spectrum of blue-emitting exo-AuNCs, which has only Au(0) peak (gray dotted curve). (B) XPS spectrum of red-emitting exo-AuNCs, which has approximately 10% Au(I) peak (black dotted curve) and 90% Au(0) peak (green dotted curve).
